# Egr2 and 3 Inhibit T-bet–Mediated IFN-γ Production in T Cells

**DOI:** 10.4049/jimmunol.1602010

**Published:** 2017-04-28

**Authors:** Randeep Singh, Tizong Miao, Alistair L. J. Symonds, Becky Omodho, Suling Li, Ping Wang

**Affiliations:** * The Blizard Institute, Barts and The London School of Medicine and Dentistry, Queen Mary University of London, London E1 2AT, United Kingdom; and; † Bioscience, Brunel University London, London UB8 3PH, United Kingdom

## Abstract

T-bet is important for differentiation of cytotoxic CD8 and Th1 CD4 T cells. We have discovered that Egr2 and 3 are potent inhibitors of T-bet function in CD4 and CD8 effector T cells. Egr2 and 3 were essential to suppress Th1 differentiation in Th2 and Th17 conditions in vitro and also to control IFN-γ–producing CD4 and CD8 T cells in response to virus infection. Together with Egr2 and 3, T-bet is induced in naive T cells by Ag stimulation, but Egr2 and 3 expression was inhibited by Th1–inducing cytokines. We found that Egr2 and 3 physically interact with the T-box domain of T-bet, blocking T-bet DNA binding and inhibiting T-bet–mediated production of IFN-γ. Thus, Egr2 and 3 are antagonists of T-bet function in effector T cells and are important for the control of inflammatory responses of T cells.

## Introduction

T cells are specifically activated by Ags, but they acquire diverse functions based on external signals encountered in the microenvironment that drive functional differentiation of CD8 T cells into cytotoxic T cells and CD4 T cells into different Th subsets with distinct functions (1, 2). The differentiation of T cells into different functional groups is mediated by lineage-specifying transcription factors (1, 2). T-bet is one of the essential transcription factors for the development of cytotoxic CD8 cells and Th1 cells in response to virus infection (3, 4). It induces expression of functional genes involved in effector responses, such as Gmzb and IFN-γ in CD8 T cells and IFN-γ in Th1 cells (3, 4). Although T-bet–mediated differentiation of effector cells is essential for immune responses to infection, its function is regulated to limit immunopathology driven by effector T cells and to allow the development of memory T cells (5). A number of mechanisms that regulate the function of T-bet in differentiation of effector T cells have been discovered, such as those involving Id3 and Tcf1, which counteract CD8 effector T cell differentiation (6, 7), whereas Blimp-1 cooperates with T-bet in CD8 effector differentiation (8). In Th differentiation, T-bet function is repressed in T follicular helper (Tfh), Th2, and Th17 cells by Bcl6-, GATA3-, and RORγt-mediated programs, respectively (9), whereas Runx1 and Runx3 are cofactors that promote T-bet–mediated IFN-γ production in CD4 T cells (10, 11). These counter-regulatory mechanisms drive lineage plasticity under specific differentiation conditions. However, it is unknown whether there is a general repressive mechanism that controls T-bet–mediated effector T cell differentiation.

Egr2 and 3 are zinc finger transcription factors with important roles in the development of NKT cells and self-tolerance (12–15). Previously, we have shown that Egr2 and 3 are essential for the control of the self-tolerance and inflammatory responses of effector phenotype T cells under homeostatic conditions (16). Egr2 and 3 deficiency results in excessive production of effector cytokines, such as IFN-γ, by CD4 and CD8 T cells in response to TCR stimulation (16), indicating that Egr2 and 3 are potent regulators of effector T cell differentiation and IFN-γ production. However, in contrast to our findings, it has recently been reported that Egr2 is important for T-bet expression and IFN-γ production in effector T cells (17).

In this study, we assessed the mechanisms of Egr2 and 3 function in the regulation of effector cell differentiation in response to viral infection and induction of Th differentiation, with a specific focus on the effect on T-bet function in the regulation of IFN-γ production. We demonstrate that Egr2 and 3 are not required for T-bet expression but act as inhibitors that potently suppress T-bet function in effector T cells. We discovered that Egr2 and 3 expression is inhibited by Th1-inducing cytokines in CD4 and CD8 T cells. Egr2 and 3 blocked T-bet DNA binding by physically interacting with the T-box domain of T-bet, resulting in inhibition of T-bet–mediated IFN-γ production. Thus, our findings demonstrate that Egr2 and 3 regulate the function of effector T cells by directly inhibiting T-bet, and this repressive function is counter-regulated by effector cytokines that may be important for a balanced and optimal adaptive immune response.

## Materials and Methods

### Mice

CD2-specific Egr2^−/−^ mice were established by crossing CD2^cre^ and Egr2^flox^ mice, whereas CD2-specific Egr2/3^−/−^ mice were bred by crossing CD2-specific Egr2^−/−^ with Egr3^−/−^ mice. All of these models were described previously (16). C57BL/6 mice (Charles River Laboratories) were used as controls in all experiments. All mice were used according to established institutional guidelines under the authority of a U.K. Home Office project license.

### Abs and flow cytometry

FITC-conjugated Abs to IL-2, CD4, and CD8; PE-conjugated Abs to CD4, CD8, and CD62L; PerCP-labeled Ab to CD44; allophycocyanin-conjugated Abs to IL-2, CD44, and IL-4; PE-Cy7–conjugated Abs to CD44, and IFN-γ were obtained from BD Biosciences. Abs to CD3 (clone 145-2C11) and CD28 (clone 37.51) for stimulation were purchased from BD Biosciences. FITC-conjugated Ab to IFN-γ; PE-conjugated Abs to IL-4, IL-17A, and Egr2; allophycocyanin-conjugated anti-Egr2; and PEcy7-conjugated anti–T-bet were from e-Bioscience. Anti-Myc and anti-Flag Abs were from Cell Signaling Technology. For flow cytometry analysis, single-cell suspensions were analyzed on a FACSCanto (BD), and the data were analyzed using FlowJo software (Tree Star). Cell sorting was performed on a FACSAria sorter with DIVA option (BD).

### Cell isolation and stimulation

Naive CD4 T cells were isolated by FACS of CD4^+^CD25^−^CD44^low^CD62L^+^ T cells. Purified CD4^+^ T cells were stimulated with plate-bound anti-CD3 (5 μg/ml) and anti-CD28 (2 μg/ml) Abs, in the presence or absence of IL-12 (100 ng/ml) or IFN-γ at different concentrations for the indicated times. Th differentiation was carried out by incubation of naive CD4 T cells with 5 μg/ml anti-CD3 and 2 μg/ml anti-CD28, together with 20 ng/ml IL-2 (402-ML-020; R&D Systems) for Th0; 20 ng/ml IL-2, 20 ng/ml IL-12 (419-ML-010; R&D Systems), 10 μg/ml anti–IL-4 (504102; BioLegend) for Th1; 20 ng/ml IL-2, 100 ng/ml IL-4 (574302; BioLegend), 10 μg/ml anti–IL-12 (505303; BioLegend), 10 μg/ml anti–IFN-γ (505702; BioLegend) for Th2; and 2 ng/ml TGF-β (240-B-002; R&D Systems), 100 ng/ml IL-6 (575702; BioLegend), 10 μg/ml anti–IL-4, and 10 μg/ml anti–IFN-γ for 5 d for Th17. For cytokine-producing cells, the cultures were stimulated for 3 h with 100 ng/ml PMA, 200 ng/ml ionomycin, and GolgiStop (BD) and then analyzed by intracellular cytokine staining. Egr2 and T-bet staining was analyzed using a Foxp3 staining kit (eBioscience).

### Generation of constructs

All PCR-cloning steps were performed with Pfx polymerase, and all constructs were confirmed by sequencing. Flag-tagged mouse Egr2 in the pcDNA3.1-iresGFP vector was described previously (16). Flag-tagged mouse Egr3 was created by PCR with the following primers: 5′-GCTTCGAATTCGCCACCATGGACTACAAAGACGATGACGACAAGACCGGCAAACTCGCCGAG-3′ and 5′-GGGGCGGATCCTCAACCGGTGGCGCAGGTGG-3′. The PCR product was digested with EcoRI and BamHI and inserted into the pcDNA3.1 ires-GFP vector. The T-bet–retrovirus construct was a gift from Prof. K.M. Murphy. Myc-tagged T-bet and Myc-tagged T-box were cloned from this construct by PCR with the following primers: Myc-T-bet, sense: 5′-CTAGACTCGAGGCCACCATGGGCATCGTGGAGCCGGGC-3′ and antisense: 5′-TGGTGGGAATTCTCACAGATCCTCTTCTGAGATGAGTTTTTGTTCGCCGTTGGGAAAATAATTATA-3′ and Myc–T-box, sense: 5′-CTAGACTCGAGGCCACCATGAAGCTGAGAGTCGCGC-3′ and antisense: 5′-GGTGGAATTCTCACAGATCCTCTTCTGAGATGAGTTTTTGTTCGCCGTTCTCCCGGAATCC-3′. PCR products were subcloned into the pcDNA3.1-ires-GFP vector via XhoI and EcoRI sites.

### Cellular transfection and coprecipitation

HEK293 cells (American Type Culture Collection) were transfected with Flag-tagged mouse Egr2, Flag-tagged Egr3, Myc-tagged T-bet, or Myc-tagged T-box, either singly or in various combinations, by calcium precipitation. The expression of each tagged protein was confirmed by immunoblotting with Abs against Myc for T-bet and T-box or Flag for Egr2 and Egr3 (Cell Signaling Technology). Coprecipitation was carried out by precipitating T-bet or Egr2 with anti-Myc or anti-Flag Abs, respectively. The precipitates were then immunoblotted with the reciprocal Abs, as indicated. For primary cells, coprecipitation was carried out by precipitating with anti–T-bet or anti-Egr2 and then immunoblotting with the reciprocal Abs, as indicated.

### IFN-γ reporter assay

The IFN-γ reporter construct was generated using the same strategy as in Hatton et al. (18) by cloning the CNS-22 enhancer region upstream of the −468 bp fragment of the mouse IFN-γ promoter into the pgl2b basic luciferase vector (Promega, Madison, WI) using primers CNS-22 sense: 5′-TACCGAGCTCCATGCAAGTATTAACATGC-3′ and antisense: 5′-CTTTTAGATCTGAAAATTAGACACCTAATTTGC-3′ and −468 sense: 5′-TTTTCAGATCTAAAAGTTTGAAAAGGCTTCCCCC-3′ and antisense: 5′-ATGCCAAGCTTTGTCTTCTCTAGGTCAGCCG-3′.

A total of 5–10 × 10^6^ EL4 cells (ATCC) was electroporated using the Gene Pulser II (Bio-Rad) at 260 V and 960 mF with 0.4 μg of the indicated constructs and 50 ng of pRL-TK. Twenty-four hours later, transfected cells were lysed in Passive Lysis Buffer, and a Dual Luciferase Assay (Promega) was performed by reading the relative light units of the lysate with the firefly substrate and the *Renilla* substrate with a KC4 luminometer (Bio-Tek Instrument). Each transfection was performed in triplicate, and data are from a minimum of three independent experiments.

### EMSA

The consensus probe for T-bet (5′-GACAGCTCACACTGGTGTGGAGCAGGG-3′) was labeled with Cyanine 5 (Sigma-Aldrich) and used in binding reactions with nuclear extracts from CD4 T cells stimulated with anti-CD3 and anti-CD28 for 16 h and then restimulated with PMA and ionomycin for 30 min. For supershift reactions, anti–T-bet (H-210; Santa Cruz Biotechnology) was added after 10 min of incubation. The samples were electrophoresed on 5% polyacrylamide gels in 0.5× TBE, and the gels were scanned using a Typhoon 9400 imager (Amersham Biosciences).

### Chromatin immunoprecipitation assays

A total of 5 × 10^6^ CD4 cells from infected and uninfected mice was cross-linked with 1% formaldehyde for 10 min at room temperature. After quenching of formaldehyde with 125 mM glycine, chromatin was sheared by sonication using a Bioruptor Pico sonication system (Diagenode). The fragmented chromatin was ∼200–1000 bp, as analyzed on agarose gels. After preclearing, chromatin (500 μg) was subjected to immunoprecipitation with specific anti–T-bet Ab (H-210; Santa Cruz Biotechnology), or with anti-Ig as negative control, at 4°C overnight. Immunocomplexes were recovered by incubation with Protein G beads. DNA was purified using a QIAquick PCR Purification Kit (QIAGEN) and used as a template for PCR amplification, with specific primers flanking the T-bet binding sites in the CNS enhancer regions of the *Ifng* locus, as defined previously (18, 19). The following primers (20) were used: CNS-34 (−33,884 to −33,757): sense 5′-TTTGGTTGGCTTACTCACTTTATTC-3′ and antisense 5′-CATCGAGGTGCCATTAACTATCA-3′; CNS-22 (−21,836 to −21,724): sense 5′-GGTGATCCACAGGAAGGAGA-3′ and antisense 5′-GAGCAGAAATTTGGCCTCTT-3′; and CNS+30 (+29,686 to +29,755): sense 5′-TCCGACGAGTGACCAAGATG-3′ and antisense 5′-CCCCAGCGGCTCTCTAAAG-3′.

Chromatin immunoprecipitation data are presented as the percentage of input DNA.

### Quantitative real-time PCR

Total RNA was extracted from cells using TRIzol Reagent (Invitrogen) and reverse transcribed using random primers (Invitrogen). Quantitative real-time PCR was performed on a Rotor-Gene system (Corbett Robotics) using SYBR Green PCR Master Mix (QIAGEN). The following primers were used: Egr2 sense 5′-TCAATGTCACTGCCGCTGAT-3′ and antisense 5′-AGAAATGATCTCTGCAACCAGAA-3′, Egr3 sense 5′-GATCCACCTCAAGCAAAAGG-3′ and antisense 5′-CGGTGTGAAAGGGTGGAAAT-3′, T-bet sense 5′-CATTGCAGTGACTGCCTACC-3′ and antisense 5′-CACTCGTATCAACAGATGCG-3′, IFN-γ sense 5′-CCATCAGCAACAACATAAGC-3′ and antisense 5′-AGCTCATTGAATGCTTGGCG-3′, and GAPDH sense 5′-TGCACCACCAACTGCTTAGC-3′ and antisense 5′-GGCATGGACTGTGGTCATGAG-3′. The data were analyzed using Rotor-Gene software. All samples were run in triplicate, and relative mRNA expression levels were obtained by normalizing against the level of Gapdh from the same sample under the same program using this equation: relative expression = 2^(CTgapdh − CTtarget).

### Viruses and infection

Vaccinia virus (Western Reserve strain of vaccinia virus [VV_WR_]) stocks (21) were grown using TK143 cells in T175 flasks, infected at a multiplicity of infection of 0.5. Cells were harvested at 72 h, and virus was isolated by rapidly freeze-thawing the cell pellet three times in 5 ml of DMEM containing 10% FCS, as previously described (21). Cell debris was removed by centrifugation. Clarified supernatant was frozen at −80°C as virus stock. VV_WR_ stocks were titrated using TK143 cells.

LB27.4 cells are B cell hybridoma cells that express MHC class I and II proteins of the H-2b haplotype (22). These cells were maintained in DMEM supplemented with 10% FCS, 2 mM l-glutamine, and antibiotics. Cells were routinely tested for mycoplasma contamination. Cells were infected with VV_WR_ at a multiplicity of infection ∼ 5 and harvested between 8 and 12 h postinfection. Cells were then resuspended in complete RPMI 1640 and used as APCs for stimulation of viral-specific T cells in vitro.

Mice were infected i.p. with 4 × 10^6^ PFU or intranasally with 3 × 10^5^ PFU vaccinia virus in 100 μl or 10 μl of physiological saline, respectively.

### Statistics

A two-tailed nonparametric Mann–Whitney *U* test, as implemented in the R package coin (23), was used to analyze the statistical significance of differences between groups. Differences with a *p* value <0.05 were considered significant.

## Results

### Egr2 and 3 are coexpressed with T-bet in T cells and control IFN-γ production

**FIGURE 1.**
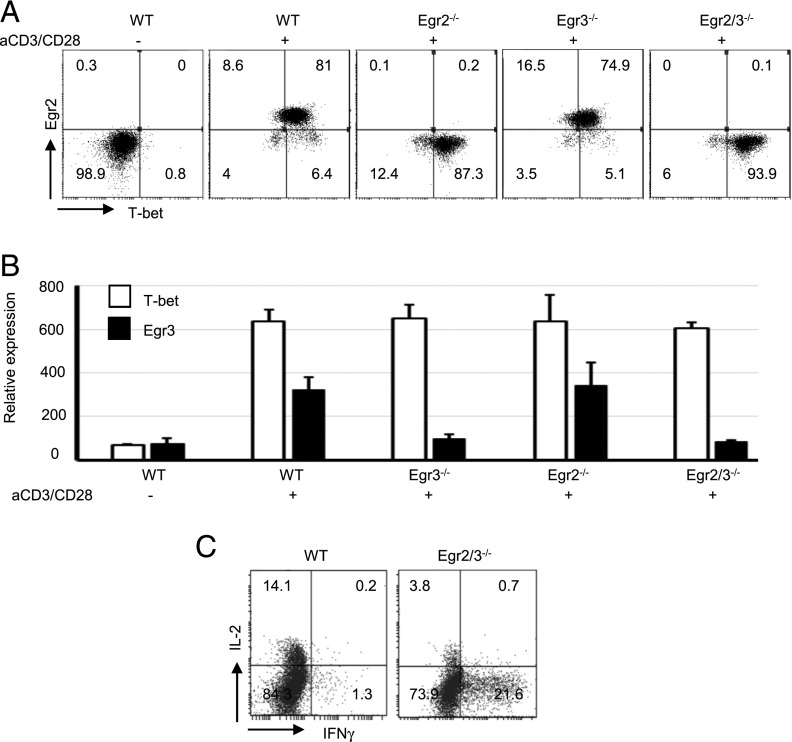
Egr2 is coexpressed with T-bet in activated T cells and, together with Egr3, controls the production of IFN-γ. (**A**) Expression of T-bet and Egr2 in CD4 T cells from WT, CD2-specific Egr2-deficient (Egr2^−/−^), Egr3-deficient (Egr3^−/−^), and CD2-specific Egr2 and 3–deficient (Egr2/3^−/−^) mice after stimulation with anti-CD3 and anti-CD28 for 16 h. (**B**) T-bet and Egr3 expression was analyzed by RT-PCR. Relative expression was calculated against GAPDH. (**C**) IL-2 and IFN-γ–producing T cells. All data are representative of three independent experiments.

Egr2 and 3 are important for maintaining the homeostasis of T cells (16, 24). Defects in Egr2 and 3 lead to the development of autoimmune disease with high levels of IFN-γ–producing CD4 T cells (16). IFN-γ is the signature cytokine for effector CD4 and CD8 T cells in adaptive immune responses, and T-bet is one of the key transcription factors for inducing IFN-γ expression (1, 2). In contrast to our previous results (16, 24), recent findings suggest that Egr2 is important for T-bet expression and IFN-γ production by effector T cells in response to infection (17). To assess the roles of Egr2 and 3 in IFN-γ production in T cells, naive CD4 T cells were stimulated in vitro with anti-CD3 and anti-CD28, and the expression of T-bet and IFN-γ was analyzed. We found that Egr2 was coinduced with T-bet in effector T cells from wild-type (WT) mice in response to TCR stimulation in vitro (Fig. 1A). Single defects in Egr2 or 3, as well as Egr2 and 3 double deficiency, did not alter the expression of T-bet (Fig. 1A). T-bet and Egr3 expression was also assessed by RT-PCR, and a similar pattern of T-bet expression was detected (Fig. 1B). Although T-bet expression was not altered, the proportion of IFN-γ–producing T cells among Egr2 and Egr3–deficient T cells was profoundly increased following TCR stimulation (Fig. 1C).

**FIGURE 2.**
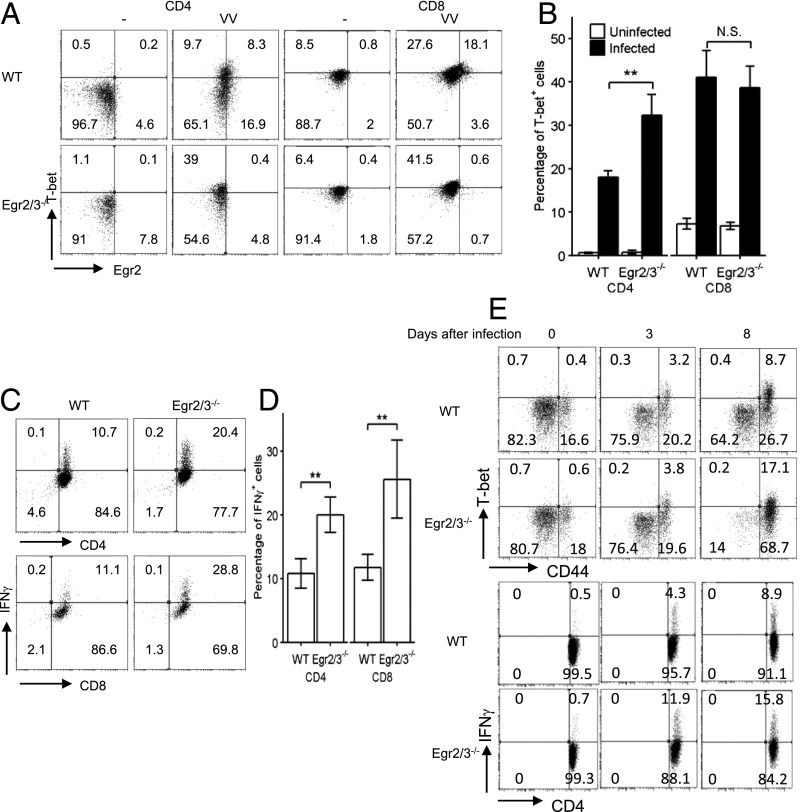
Egr2 and 3 are important for the control of IFN-γ production by T cells in response to viral infection. WT and CD2-specific Egr2 and 3–deficient (Egr2/3^−/−^) mice were infected with VV_WR_ (i.p.) for 7 d. (**A** and **B**) T-bet and Egr2–expressing CD4 and CD8 T cells before infection and postinfection. (**C** and **D**) Splenic CD4 and CD8 cells from viral-infected mice were incubated with virus-infected LB27.4 cells for 16 h before the analysis of IFN-γ–producing cells (**E**). T-bet, CD44, and IFN-γ expression in CD4 T cells on days 0, 3, and 8 postinfection. The data in (A), (C), and (E) are from pooled cells from five mice in each group and are representative of at least three independent experiments. Data in (B) and (D) are mean ± SD. **p < 0.01, Mann–Whitney two-tailed test. N.S., *p* > 0.05.

To further assess the effect of Egr2 and 3 deficiency on T-bet expression in effector T cells induced by viral infection, WT and CD2-Egr2/3^−/−^ mice were infected i.p. with VV_WR_. Seven days postinfection, the expression of Egr2 and T-bet and IFN-γ production in T cells were analyzed. In response to infection, T-bet and Egr2 were induced in CD4 and CD8 T cells (Fig. 2A, 2B). A proportion of T-bet^+^ CD4 and CD8 T cells coexpressed Egr2 (Fig. 2A). T-bet expression was not defective in CD4 and CD8 T cells from CD2-specific Egr2/3^−/−^ mice in response to virus infection; indeed, T-bet^+^ CD4 T cells were higher in CD2-specific Egr2/3^−/−^ mice (Fig. 2A, 2B). IFN-γ–producing cells among CD4 and CD8 T cells were higher in CD2-specific Egr2/3^−/−^ mice (Fig. 2C, 2D). To assess the possibility that the high levels of T-bet^+^ CD4 cells in CD2-specific Egr2/3^−/−^ mice are due to hyperactivation of T cells at late stages of infection, the kinetics of T-bet and IFN-γ expression postinfection was analyzed. T-bet was expressed in CD4^+^CD44^hi^ cells at similar levels in WT and CD2-specific Egr2/3^−/−^ mice on day 3 postinfection, whereas IFN-γ–producing cells were higher in CD2-specific Egr2/3^−/−^ mice (Fig. 2E). Eight days postinfection, an increase in T-bet– and IFN-γ–expressing CD4 T cells was detected in CD2-specific Egr2/3^−/−^ mice (Fig. 2E), suggesting that the high levels of T-bet–expressing T cells in CD2-specific Egr2/3^−/−^ mice result from hyperactivation of T cells at late stages of infection. Taken together, these data demonstrate that Egr2 and 3 are not required for T-bet expression, but they do control the production of IFN-γ by effector T cells.

### Egr2 and 3 control Th1 differentiation of CD4 T cells

**FIGURE 3.**
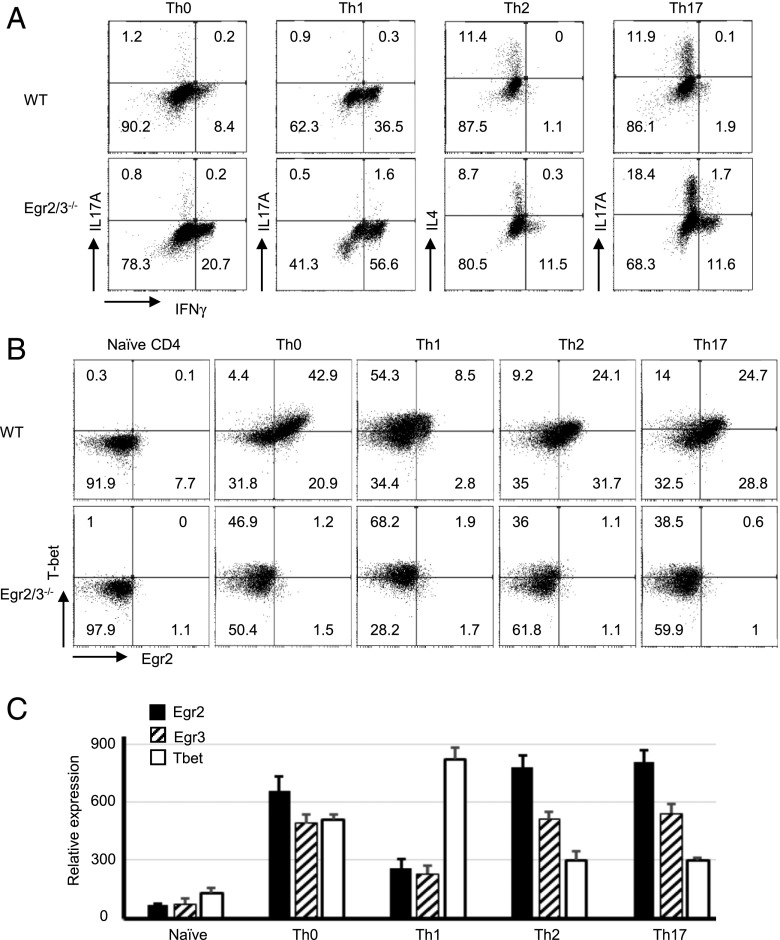
Egr2 and 3 control IFN-γ in a lineage-independent fashion. CD4 cells isolated from WT and CD2-specific Egr2 and 3–deficient (Egr2/3^−/−^) mice were cultured in vitro under the indicated Th conditions in the presence of anti-CD3 and anti-CD28 for 5 d before analysis of cytokine-producing cells (**A**) and the expression of Egr2 and T-bet (**B**) by flow cytometry, as well as by RT-PCR for WT T cells (**C**). Data are representative of three independent experiments. Data in (C) are mean ± SD.

IFN-γ is the signature cytokine for Th1 cells, whereas T-bet is the lineage-specifying transcription factor for Th1 differentiation and is important for IFN-γ expression (2). To investigate whether Egr2 and 3 affect the differentiation of Th1 cells, naive CD4 cells were isolated from WT and CD2-specific Egr2/3^−/−^ mice and cultured under different Th conditions in vitro. In unpolarized or Th1-polarized conditions, IFN-γ–producing cells were highly increased among Egr2 and Egr3–deficient CD4 cells (Fig. 3A). Egr2 and 3 deficiency did not affect Th2 differentiation and modestly enhanced differentiation of Th17 cells (Fig. 3A), which is consistent with our previous findings (25). Interestingly, under Th2 and Th17 conditions, a proportion of IFN-γ–producing cells was detected among Egr2 and 3–deficient CD4 cells (Fig. 3A). Notably, most of the IFN-γ–producing Egr2 and 3–deficient cells under Th2 and Th17 conditions did not coproduce IL-4 or IL-17 (Fig. 3A), indicating that Egr2 and 3 suppress Th1-like differentiation; however, Egr2 and 3 deficiency does not skew the differentiation of Th2 and Th17 cells to Th1.

T-bet was differentially induced in different Th conditions. It was highly induced in Th1 cells but was expressed at a low level in Th2 and Th17 cells in WT cultures (Fig. 3B). Egr2 and 3 deficiency did not alter the expression of T-bet following differentiation (Fig. 3B). Under Th0, Th2, and Th17 conditions, the majority of T-bet^+^ cells also expressed Egr2 (Fig. 3B). However, in Th1 conditions, despite high levels of T-bet, only a small population of T-bet–expressing Th1 cells coexpressed Egr2 (Fig. 3B). RT-PCR analysis showed that Egr2 and 3 have a similar expression pattern in different Th subsets, and the differences in T-bet expression in Th subsets were also similar to those analyzed by flow cytometry (Fig. 3C). These results demonstrate that, although T-bet is highly induced in Th1 cells, it is also expressed in Th2 and Th17 conditions, which suggest that coexpression of Egr2 with T-bet in these conditions may contribute to the repression of T-bet–mediated production of Th1 cytokines, such as IFN-γ. If Egr2 and/or 3 have a repressive effect on T-bet–mediated Th1 differentiation, the limited induction of Egr2 in Th1 conditions may be important for optimal Th1 differentiation.

### Suppression of Egr2 expression in T cells by Th1-inducing cytokines

**FIGURE 4.**
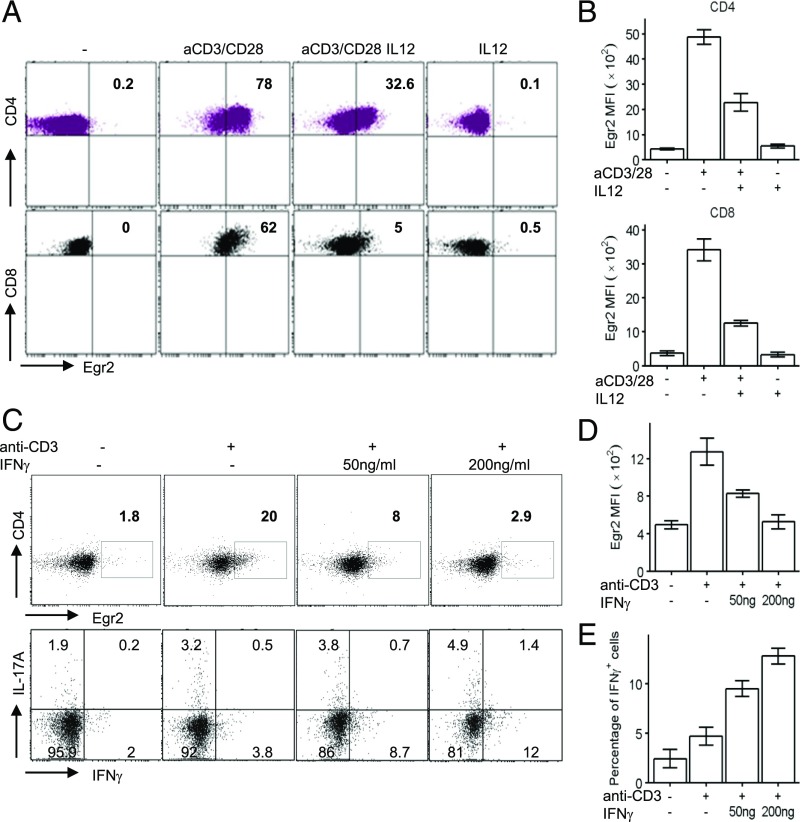
Expression of Egr2 is suppressed by IL-12 and IFN-γ. (**A** and **B**) CD4 and CD8 T cells from WT mice were stimulated as indicated for 16 h. Expression of Egr2 was analyzed by flow cytometry (A), and the mean fluorescence intensity (mean ± SD) from five replicated samples was analyzed (B). (**C**) Naive CD4 T cells from WT mice were stimulated as indicated for 16 h. Expression of Egr2 (upper panels) and IFN-γ and IL-17A–producing cells (lower panels) were analyzed. Mean ± SD of three replicated samples for Egr2 mean fluorescence intensity (**D**) and percentage of IFN-γ–producing cells (**E**). The data are representative of two independent experiments.

Th1 cytokines are essential for adaptive and innate immune responses. T-bet has been found to be induced by Ag and Th1 cytokine stimulation in CD4 and CD8 T cells (1, 2, 4). The reduced expression of Egr2 in Th1 cells compared with Th2 and Th17 cells (Fig. 3B) may indicate that Egr2 is downregulated in T cells under Th1 conditions. To investigate the effect of Th1-inducing cytokines on the expression of Egr2, CD4 and CD8 T cells from wild-mice were stimulated with anti-CD3 and anti-CD28 in the presence or absence of IL-12. The induction of Egr2 was inhibited by IL-12 (Fig. 4A, 4B), suggesting that Egr2 expression is inhibited in Th1-inducing microenvironments, which may be important for optimal differentiation of effector CD8 and Th1 cells. Furthermore, IFN-γ also suppressed the expression of Egr2, and the reduction in Egr2 expression by IFN-γ was accompanied by induction of IFN-γ–producing cells in response to TCR stimulation (Fig. 4C–E), which suggests that Th1 cytokines serve as feedback repressors of Egr2 expression in Th1 cells.

### Egr2 and 3 directly inhibit T-bet function

**FIGURE 5.**
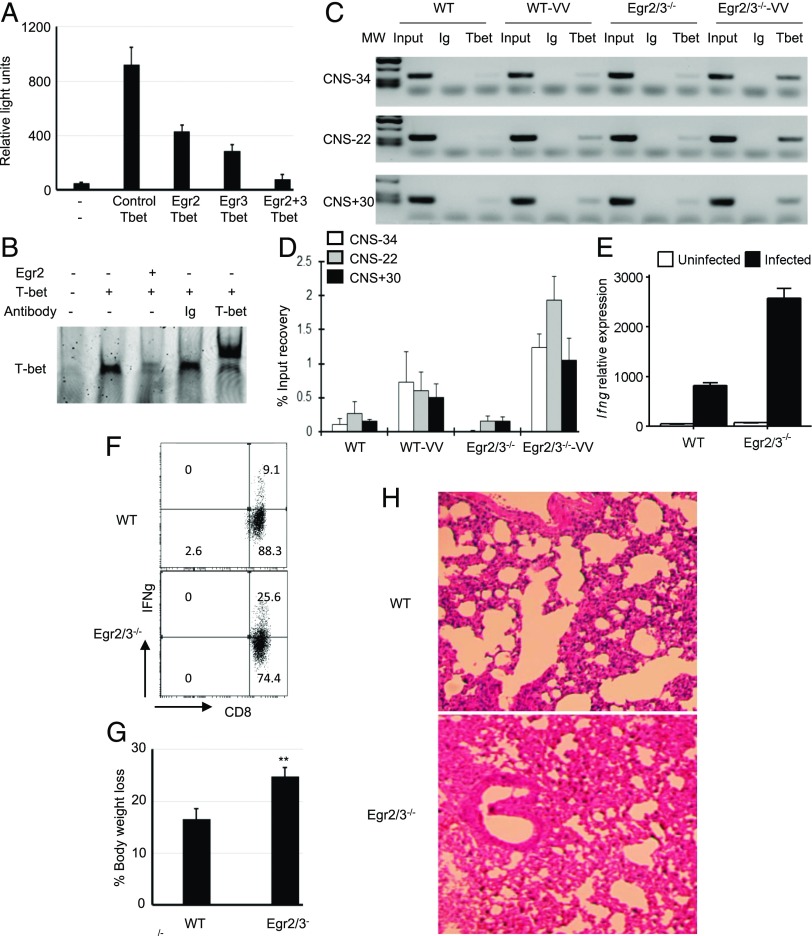
Egr2 and 3 suppress T-bet–mediated IFN-γ reporter gene activity and block interaction of T-bet with its DNA binding site. (**A**) EL4 cells were cotransfected with an IFN-γ reporter gene (18) and the indicated genes. Twenty-four hours after transfection, cells were analyzed for reporter gene activity. (**B**) HEK cells were transfected with T-bet, with or without cotransfection of Egr2. The nuclear lysates from transfected cells were used in an EMSA for detection of the interaction of T-bet with its consensus DNA oligonucleotide. The specificity was confirmed by supershift with an anti–T-bet Ab. (**C**–**E**) WT and CD2-specific Egr2 and 3–deficient (Egr2/3^−/−^) mice were infected with VV_WR_ (i.p.) for 7 d. (C) A T-bet chromatin immunoprecipitation assay was performed to detect interaction of T-bet with identified enhancer regions of the *Ifng* locus CNS-34, CNS-22, and CNS+30 (18, 19) in CD4 T cells isolated from spleens and lymph nodes of infected mice. (D) Real-time PCR measurement of T-bet binding to the *Ifng* locus, as in (C), presented as the percentage of input DNA. (E) RT-PCR analysis of *Ifng* expression in CD4 T cells isolated from spleens and lymph nodes of viral-infected mice. (**F**–**H**) WT and Egr2/3^−/−^ mice were infected with VV_WR_ intranasally for 7 d. (F) IFN-γ–producing CD8 T cells from lung tissues of infected WT and Egr2/3^−/−^ mice. (G) Percentage of body weight loss 7 d postinfection. (H) H&E staining of lung tissues 7 d postinfection (original magnification ×10). Data in (A)–(E) are from pooled cells from five mice of each group and are representative of three independent experiments. Data in (F)–(H) are representative of three independent experiments, with four or five mice per group. Data in (A)–(F) are the mean ± SD. **p < 0.01 versus WT, Mann–Whitney two-tailed test.

The coexpression of Egr2 and 3 with T-bet and the excessive production of IFN-γ by Egr2 and 3–deficient T cells suggest a regulatory role for Egr2 and 3 in T-bet–mediated IFN-γ expression. To investigate this, a T-bet–dependent IFN-γ reporter, consisting of the proximal IFN-γ promoter flanked by the −22-kb enhancer region (18), was analyzed in the presence or absence of Egr2 or 3. In the absence of T-bet, reporter activity was barely detected, whereas T-bet induced high levels of reporter gene expression (Fig. 5A), consistent with previous reports (18). T-bet–mediated reporter activity was profoundly inhibited in the presence of Egr2 or 3 and was completely abolished by cotransfection of Egr2 and 3 (Fig. 5A). These findings demonstrate that, although Egr2 and 3 are not required for the expression of T-bet, they directly inhibit T-bet–mediated IFN-γ expression. Next, we analyzed the DNA binding of T-bet in the presence or absence of Egr2 and found that Egr2 blocked interaction of T-bet with its DNA binding site (Fig. 5B).

To further address the impact of Egr2 on T-bet–mediated IFN-γ production in adaptive immune responses, CD4 T cells isolated from WT and CD2-Egr2/3^−/−^ mice 7 d postinfection with VV_WR_ virus were analyzed for interaction of T-bet with its binding sites in the −34-kb, −22-kb, and +30-kb enhancer regions of the *Ifng* locus (CNS-34, CNS-22, and CNS+30) (18, 19). The binding of T-bet to the CNS-34, CNS-22, and CNS+30 enhancer regions of the *Ifng* locus was increased in Egr2/3-deficient CD4 T cells compared with WT counterparts in response to virus infection (Fig. 5C, 5D). Consistent with the increased binding of T-bet to the *Ifng* locus, the expression of *Ifng* was also profoundly increased in Egr2 and 3–deficient CD4 T cells (Fig. 5E). In addition to the increased IFN-γ–producing T cells in peripheral lymphoid organs in viral-infected CD2-Egr2/3^−/−^ mice (Fig. 2E), IFN-γ–producing CD8 T cells from infected lung tissue of CD2-Egr2/3^−/−^ mice were significantly increased in comparison with WT mice (Fig. 5F). CD2-Egr2/3^−/−^ mice had more severe infection and inflammatory pathology in the lungs than did WT mice (Fig. 5G, 5H). Thus, Egr2 and 3 are important for the control of T-bet–mediated IFN-γ production in antiviral responses, which may be important for the control of immunopathology.

### Inhibition of T-bet function is due to physical interaction with Egr2 and 3

**FIGURE 6.**
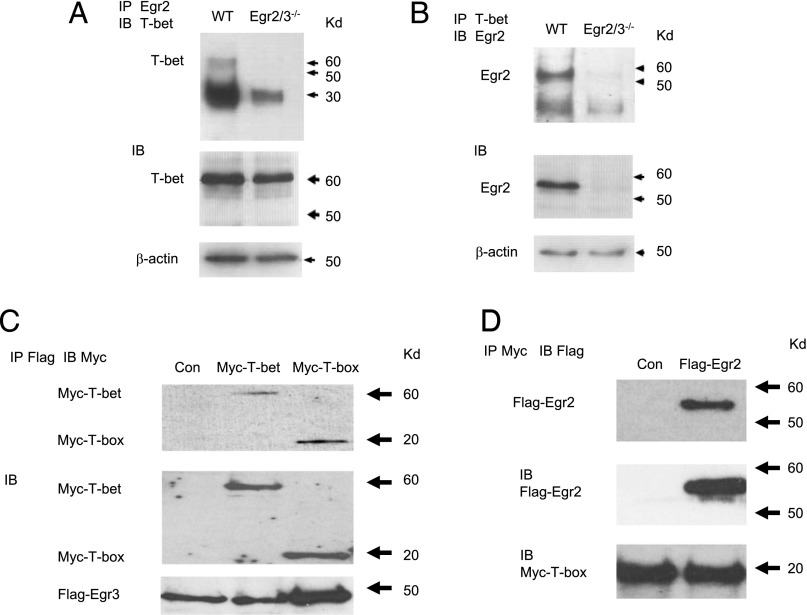
Egr2 and 3 physically interact with T-bet in T cells. (**A** and **B**) CD4 T cells were isolated from WT and CD2-specific Egr2 and 3–deficient (Egr2/3^−/−^) mice and stimulated in vitro with anti-CD3 and anti-CD28 for 16 h. The nuclear lysates were precipitated with anti-Egr2 (A) or anti-T-bet (B). The precipitates were blotted with anti–T-bet (A) or anti-Egr2 (B) Ab. Part of each lysate was blotted directly with anti–T-bet (A), anti-Egr2 (B), or anti-actin (A and B) as loading controls. (**C**) HEK cells were cotransfected with Flag-tagged Egr3 and either full-length Myc-tagged T-bet or the Myc-tagged T-box domain. The nuclear lysates were precipitated with anti-Flag Ab, and the precipitates were blotted with anti-Myc Ab (top panel). Expression of transfected proteins was confirmed by blotting of lysates with anti-Myc and anti-Flag Abs (middle and bottom panel, respectively). (**D**) HEK cells were cotransfected with Flag-tagged Egr2 and the Myc-tagged T-box domain. The nuclear lysates from transfected cells were precipitated with anti-Myc Ab, and the precipitates were blotted with anti-Flag Ab (top panel). Protein expression was confirmed by blotting of lysates with anti-Flag and anti-Myc Abs (middle and bottom panel, respectively). The data are representative of three to five experiments.

Egr2 and 3 do not directly regulate the expression of T-bet, but they inhibit T-bet–mediated expression of IFN-γ. To assess whether Egr2 physically interacts with T-bet, naive CD4 T cells from WT mice were stimulated with anti-CD3 and CD28 in vitro to induce T-bet and Egr2 expression and were analyzed for Egr2 and T-bet interaction by coimmunoprecipitation with anti-Egr2 and anti–T-bet Abs. The precipitates from each Ab were analyzed for Egr2 and T-bet. We discovered that Egr2 and T-bet were present in anti-Egr2 and anti–T-bet precipitates (Fig. 6A, 6B), demonstrating a physical interaction between Egr2 and T-bet proteins in activated T cells. We have shown that Egr2 and 3 have an overlapping function in inhibiting T-bet–mediated IFN-γ reporter activity (Fig. 5A). To investigate whether Egr3 and T-bet can also interact, Flag-tagged Egr3 and Myc-tagged T-bet were cotransfected and investigated for Egr3 and T-bet interaction by coimmunoprecipitation. Indeed, similar to Egr2, an interaction between Egr3 and T-bet was detected (Fig. 6C). We further showed that Egr2 and 3 specifically interact with the T-box domain of T-bet (Fig. 6C, 6D), which mediates DNA binding. Collectively, these results demonstrate that Egr2 and 3 block the function of T-bet in T cells by preventing its interaction with DNA binding sites, resulting in suppression of T-bet–mediated differentiation of effector CD8 and Th1 cells.

## Discussion

T-bet is one of the important transcription factors for cytotoxic CD8 and Th1 CD4 cell differentiation in response to viral infection (1, 2). T-bet function is regulated by multiple transcription factors that cooperate with or repress T-bet. Runx1 and Runx3 cooperate with T-bet, thereby promoting IFN-γ production and Th1 differentiation (10, 11), whereas transcription factors such as Gata3 and Bcl6 repress T-bet function during Th2 and Tfh differentiation, respectively (26, 27). IFN-γ expression is also suppressed in Th2 and Th17 cells by repressive chromatin modifications (28). We have now discovered a repressive function of Egr2 and 3 for inhibition of T-bet function in effector T cells that does not depend upon differentiation conditions. T-bet is induced by Ag stimulation and inflammatory cytokines in naive T cells, and increased T-bet activity has been found in autoimmune diseases (29). The inhibition of T-bet function by Egr2 and 3 may be important for the control of excessive inflammatory responses mediated by T-bet in adaptive and autoimmune responses. In contrast to a normal T-bet expression in Egr2 or Egr3 single-deficient T cells, as well as Egr2 and 3 double-deficient T cells, a recent report showed defective IFN-γ production by Egr2-deficient T cells that was attributed to direct regulation of the *Tbx21* and *Ifng* loci by Egr2 in T cells in response to viral infection (17). Although we cannot explain the differences, Egr2-knockout models from different laboratories showed normal T cell responses to infection (30), suggesting that Egr2 single deficiency does not affect the function of T cells in adaptive immune responses.

Control of T-bet function is important for differentiation of Tfh, Th2, and Th17 cells (28). Our findings demonstrate that Egr2 and 3 deficiency does not render naive CD4 T cells resistant to Th2 and Th17 differentiation, but it does result in a profound increase in IFN-γ–producing cells that are not producing IL-4 or IL-17 under Th2- or Th17-differentiation conditions, respectively. These data suggest that Egr2 and 3 provide additional control of Th1 differentiation for the cells that are not producing Th2 or Th17 cytokines, and this function may be independent of lineage-specific transcriptional networks.

Previously, we found that Egr2 and 3 control differentiation of T cells by multiple mechanisms, including induction of SOCS1 and SOCS3 expression (16), inhibition of Batf activity resulting in a reduction in Th17 differentiation (25), and Egr2 and 3 double deficiency–impaired Tfh differentiation due to impaired expression of Bcl6 (21). The impaired Bcl6 expression may also play a role in the increased Th1-type differentiation (27). Consistent with our previous report, IL-17–producing T cells were also increased among Egr2 and 3–deficient CD4 T cells under Th17 conditions. However, in contrast to the relative increase in IL-17–producing cells under Th17 conditions, our novel discovery is that IFN-γ–producing T cells among Egr2 and 3–deficient T cells were profoundly increased in all Th conditions, as well as in effector T cells generated in response to viral infection, demonstrating a distinct and fundamental role for Egr2 and 3 in the control of T-bet–mediated IFN-γ production in effector T cells. T-bet–mediated IFN-γ production is one of the key functions of NK and NKT cells, which play important roles in innate immune responses and inflammatory autoimmunity (18). Interestingly, Egr2 has been found to be important for the development of NKT cells (15), raising the possibility that the mechanism that we have discovered for T-bet inhibition may also be relevant for innate-like T cells.

Egr2 expression is lower in Th1 cells than in Th2 and Th17 cells, suggesting that feedback inhibition of Egr2 and 3 expression by IFN-γ is important for Th1 differentiation. We discovered that Th1-inducing cytokines suppress TCR-induced Egr2 expression in CD4 and CD8 T cells. Thus, Th1-inducing cytokines not only induce the T-bet–mediated Th1-differentiation program, they optimize T-bet function by suppressing Egr2 and 3 expression. However, the excessive IFN-γ production by Egr2 and 3–deficient CD4 and CD8 T cells indicates that the basal levels of Egr2 and 3 are also important to control Th1 and CD8 effector cell–mediated adaptive immune responses.

Our findings demonstrate a novel suppressive function of Egr2 and 3 on T-bet–mediated IFN-γ production by T cells, indicating that Egr2 and 3 have an important role in the control of effector T cell differentiation that may limit immunopathology.
